# Control efficacy of complex networks

**DOI:** 10.1038/srep28037

**Published:** 2016-06-21

**Authors:** Xin-Dong Gao, Wen-Xu Wang, Ying-Cheng Lai

**Affiliations:** 1 School of Systems Science, Beijing Normal University, Beijing, 100875, P. R. China; 2 Business School, University of Shanghai for Science and Technology, Shanghai 200093, China; 3 School of Electrical, Compute and Energy Engineering, Arizona State University, Tempe, Arizona, 85287 USA; 4 Department of Physics, Arizona State University, Tempe, Arizona 85287, USA

## Abstract

Controlling complex networks has become a forefront research area in network science and engineering. Recent efforts have led to theoretical frameworks of controllability to fully control a network through steering a minimum set of driver nodes. However, in realistic situations not every node is accessible or can be externally driven, raising the fundamental issue of control efficacy: if driving signals are applied to an arbitrary subset of nodes, how many other nodes can be controlled? We develop a framework to determine the control efficacy for undirected networks of arbitrary topology. Mathematically, based on non-singular transformation, we prove a theorem to determine rigorously the control efficacy of the network and to identify the nodes that can be controlled for any given driver nodes. Physically, we develop the picture of diffusion that views the control process as a signal diffused from input signals to the set of controllable nodes. The combination of mathematical theory and physical reasoning allows us not only to determine the control efficacy for model complex networks and a large number of empirical networks, but also to uncover phenomena in network control, e.g., hub nodes in general possess lower control centrality than an average node in undirected networks.

A frontier area of research in network science and engineering is controlling complex networks^1^,^2^,^3^,^4^,^5^,^6^,^7^,^8^,^9^,^10^,^11^,^12^,^13^,^14^,^15^,^16^,^17^. Nearly two decades of efforts have resulted in tremendous advances^18^,^19^,^20^,^21^,^22^,^23^ in our understanding of complex networked dynamical systems, beginning from the discoveries of the small-world^24^ and scale-free^25^ topologies in a large variety of natural, technological, and social systems. The efforts have created a knowledge foundation based on which the problem of control can be investigated. Ideally, to make controlling complex networked systems practically significant, one must consider nonlinear dynamical processes, due to the ubiquity of nonlinearity in the real world. However, to develop a general and mathematically rigorous control framework for complex networks hosting nonlinear dynamics is at present not achievable. A “stepping stone” is to consider linear dynamical processes on complex networks, an approach pioneered by Liu *et al*.^4^, who developed a framework based on Lin’s classic structural controllability theory (SCT)^26^. In particular, SCT answers the following question: given a complex, directed network, what is the minimal set of inputs (driver nodes) required to fully control the network in the sense that the entire network can be driven from an arbitrary initial state to an arbitrary final state in finite time? This was accomplished through a systematic methodology to find a minimum set of driver nodes to realize full control using the concept of maximum matching^27^,^28^,^29^. Subsequently, an exact controllability theory (ECT)^10^,^14^ was developed based on the concept of maximum multiplicity^30^ in linear algebra to identify the minimum set of driver nodes required to fully control the network. The ECT is applicable to a broader class of complex networks: weighted, directed or undirected, with or without any loop structure, etc. These efforts stimulated a great deal of interest in the linear controllability and observability framework of complex networks^5^,^6^,^7^,^8^,^9^,^10^,^11^,^12^,^13^,^14^,^15^,^16^,^17^,^31^,^32^,^33^, addressing problems such as linear edge dynamics^5^,^6^, energy cost of control^8^,^16^,^17^, the role of nodal dynamics in network controllability^34^,^35^.

In this paper, we address a fundamental and outstanding issue in controlling complex networks: *control efficacy*, the meaning of which can be understood and its significance can be appreciated, as follows. The SCT or ECT framework provides a solution of a minimum set of input signals to fully control any complex network. However, given an *arbitrary external control signal*, typically it is not possible to control the whole but only a part of the network. (The need to consider an arbitrary control signal lies in the fact that, for a network in the real world, the set of minimum input signals from the SCT or ECT framework may be physically or experimentally unrealizable.) In this regard, a related concept is control centrality^36^ derived from the SCT, which characterizes the ability of a single node to control a fraction of nodes in a directed network. Here we shall consider the more general situation where multiple input signals imposed on more than a single node are unable to control the entire network but but only a part of it, for broader classes of complex networks including weighted, undirected networks, with or without local loop structure. The main result is a control efficacy theorem and its proof, which provides a rigorous assessment of the role of an arbitrary set of nodes in partial control of the underlying network. In particular, for any given control inputs, our theorem gives the corresponding set of nodes in the network that are controllable. Our theorem of control efficacy can measure control centrality of a single node in terms of imposing a single external input signal on the node. The control centrality can be generally evaluated in arbitrary undirected networks with any distribution of link weights. We anticipate our finding to be practically significant for situations where the underlying network system is not fully accessible from the standpoint of delivering control signals. For example, for a social network, only a very limited set of nodes may be manipulated for control. For a neuronal network, only a small set of nodes can be perturbed externally. Our theory gives, in these realistic applications, a quantitative picture of what portion of the network may be controlled.

## Results

### Control efficacy of complex networks

Consider an undirected network of *N* nodes described by the following linear time invariant (LTI) dynamical system^37^,^38^,^39^:
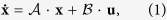
where the vector **x** ≡ (*x*_1_, …, *x*_*N*_)^T^ represents the state of all nodes at time *t*, 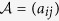 is an *N* × *N* coupling matrix of the network with the element *a*_*ij*_ representing the weight of a directed link from node *j* to node *i (a*_*ij*_ = *a*_*ji*_ for an undirected network), **u**(*t*) is the input signal of *m* controllers: **u** = (*u*_1_, …, *u*_*m*_)^T^, and 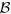 is the *N* × *m* control matrix with 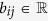 representing the strength of the input signal *u*_*j*_(*t*) on node *i*. According to the classical control theory^40^, the controllability of the system is determined completely and uniquely by the combined matrix 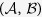. For an initial state 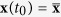, if there exists a control input **u**(*t*) that can drive the system to the final state, say **x**(*t*_1_) = 0, within the finite time interval [*t*_0_, *t*_1_], we say that the state 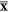 is a controllable state of the system and denote it as **x**_+_. The classic Kalman rank condition^40^ stipulates that the linear system Eq. (1) is controllable if and only if the *N* × *Nm* controllability matrix 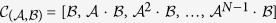 has full rank. When the system is not fully controllable or, equivalently, when the state space is not filled entirely with the controllable states **x**_+_, there can still be a controllable subspace spanned by the column vectors of the Kalman controllability matrix 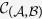. The dimension of the controllable subspace is the rank of 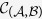: 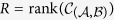, which characterizes the control efficacy of the system.

There are two difficulties in determining the rank of the controllability matrix: (1) the task is often computationally prohibitive for large networks, and (2) the controllability matrix is typically nearly singular due to the dramatic differences among its elements, making the numerical rank computation inaccurate or even divergent. We are thus led to develop a feasible and effective method to calculate the rank *R*. The starting point is a non-singular linear transformation. In particular, for an arbitrary matrix 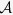 in the system equation [Eq. (1)], there exists^30^ a non-singular matrix 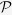 such that 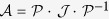 or 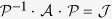 with 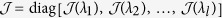, where *λ*_*i*_(*i* = 1, …, *l*) are the distinct eigenvalues of 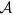 and 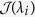 is the Jordan block matrix of 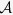 associated with the eigenvalue *λ*_*i*_. For an undirected network, the coupling matrix 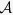 is diagonalizable and the matrix 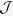 reduces to the diagonal matrix with elements being all the eigenvalues^41^.

Applying the non-singular transformations 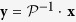 and 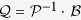, we can rewrite Eq. (1) in the following form:
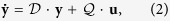
where 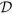 is a diagonal matrix (see Method). The controllability matrix 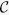 for the transformed system is
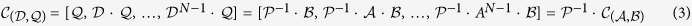


**Figure 1:**
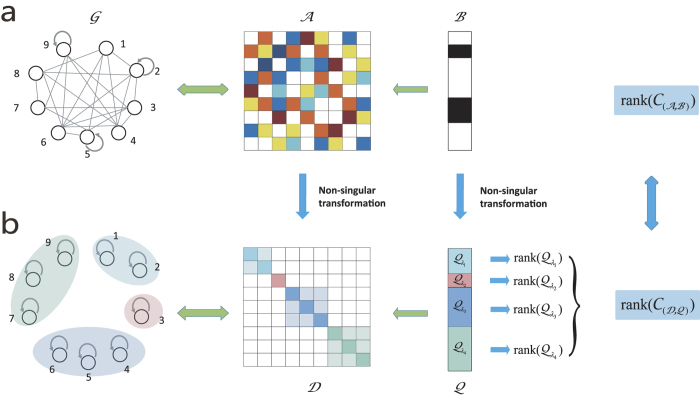
Schematic illustration of rank calculation for undirected networks with self-loops. (**a**) The adjacency matrix 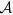 and the control input matrix 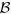 for a simple undirected network with self-loops. Each colored lattice point in 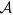 represents an element, where the colors from white to black correspond to element values from zero to one, respectively. A similar color scheme applies to the matrix 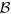. (**b**) Through a nonsingular matrix transformation, the system 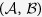 is converted into the equivalent system 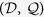, where 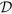 is a diagonal matrix. Distinct eigenvalues of 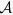 correspond to different subblocks marked with different colors. The rank of the controllability matrix for the transformed system 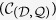 is equal to the sum of the rank values of the corresponding subblocks in the transformed control input matrix 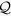, which is identical to the rank of the controllability matrix of the original system 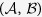.

We can verify that the systems Eqs (1) and (2) possess the same degree of controllability in the sense that 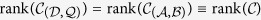, i.e., the rank of the controllability matrix of the original system is equal to 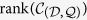, which can be calculated reliably and accurately. For an arbitrary undirected network, we can prove that 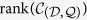 is determined by the corresponding element values in the transformed control input matrix 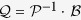 associated with the distinct eigenvalues (Method). A schematic illustration of our method to calculate the rank for undirected networks with self loops is presented in Fig. 1, and an explicit example is given in Supplementary Note 1. Our key analytic results are as follows.

#### Single control input

When there is only a single controller (i.e., when the control input matrix 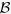 is a column vector), the task of calculating 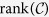 is reduced to counting the corresponding nonzero elements in the matrix 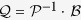. Letting the element corresponding to the eigenvalue *λ*_*i*_ be 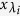 in 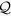, we have
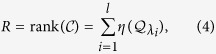
where
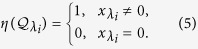


For each distinct, non-degenerate eigenvalue, the corresponding nonzero elements in 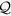 contribute equally to the value of 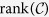. For a degenerate eigenvalue, if there are no corresponding nonzero elements in 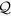, the contribution of this eigenvalue to 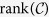 is zero. If the corresponding elements in 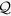 are nonzero, the degenerate eigenvalue contributes one to 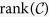 (see Method).

#### Multiple control inputs

When there are *m* control inputs, the matrix 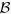 has the dimension *N* × *m*. In this case, the control efficacy is determined by the sum of the rank values of the sub-block matrices composed of the corresponding rows in the transformed control input matrix 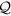 for each distinct eigenvalue (see Method). We have
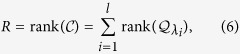


### Control Centrality

Given a complex network, it is often necessary to quantify the relative importance of the nodes with respect to a specific function. For this purpose, various kinds of centrality measures^23^ were proposed in the past, such as the degree centrality, the closeness centrality^42^, the betweenness centrality^43^, the eigenvector centrality^44^,^45^, and PageRank^46^. Control centrality has been defined in directed networks for quantifying the relative importance of nodes in effecting control^36^, i.e., if an external driver signal is applied to a node in a directed network, how many other nodes can be controlled? Our task is to extend the definition of control centrality to undirected networks and offer a control centrality measure.

Specifically, For node *i* in an undirected network, its control centrality is nothing but the dimension of the controllable subspace. When a single driving signal is applied to *i*, the corresponding control input matrix 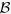 is effectively reduced to a vector **b**_(*i*)_ with a single non-zero element. For convenience, we set this element to be unity, let *R*_(*i*)_ be the rank of the controllability matrix, and rewrite the system as
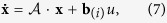
where *u* is the strength of the input signal. The value of *R*_(*i*)_ can be used to characterize node *i*’s ability to control the whole network. In Method, we provide a proof for the following inequality, which gives the upper bound of *R*_(*i*)_:
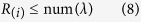
where num(*λ*) is the number of the distinct eigenvalues of the matrix 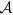. If *R*_(*i*)_ = *N*, then node *i* alone can control the whole system. However, for *R*_(*i*)_ = 1, node *i* is not able to control any other node in the networks. A value of *R*_(*i*)_ between 1 and *N* gives the dimension of the controllable subspace of node *i*. To compare the control centrality in networks with different size, the normalized control centrality *r*_(*i*)_ can be defined as the ratio of *R*_(*i*)_ to the network size *N*. Then the average value, maximum and minimum values of *r*_(*i*)_ are
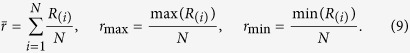


For the networks with random weights, the control centrality and the normalized control centrality can be denoted by 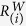 and 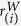 respectively.

**Figure 2:**
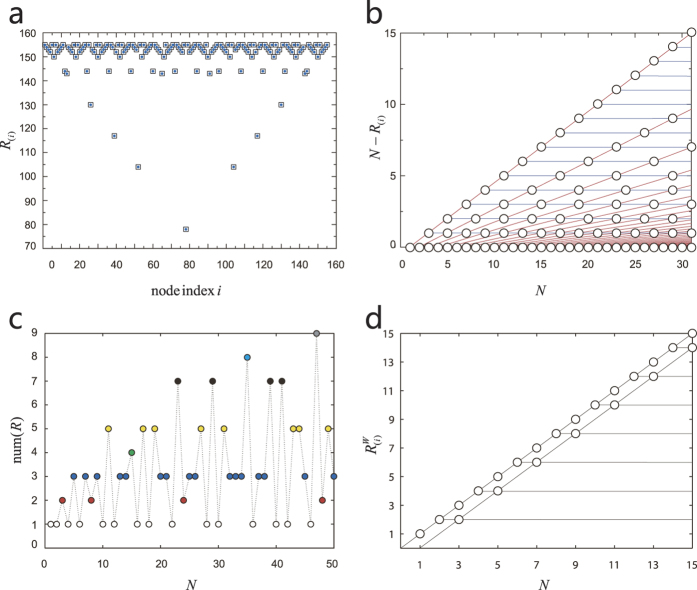
Control centrality of an undirected chain graph. (**a**) Nodal control centrality *R*_(*i*)_ versus the node index for a one-dimensional undirected chain graph of size 155, where the squares denote the results from Eq. (4) and the blue solid circles are those from Eq. (10). (**b**) All the possible values of *N* − *R*_(*i*)_ versus the system size *N*. For a fixed value of *N*, there are a finite number of *R*_(*i*)_ values. (**c**) num(*R*), the number of distinct control centralities *R*_(*i*)_ versus *N*. Each distinct value of num(*R*) is marked with a different color. It is remarkable that num(*R*) is related to the prime decomposition, as can be calculated from Eq. (11). For instance, the hollow circles represent that *N* + 1 is a prime number. (**d**) For the undirected chain graph with random weights, the corresponding control centralities 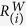 versus *N*. According to Eq. (12), there are two periodic behavior for odd and even number of nodes alternately.

We employ the criteria of control efficacy to explore undirected chains. To our surprise, complex phenomena associated with prime numbers emerge in the extremely simple regular network. Figure 2(a) shows, for an undirected chain graph of size *N* = 155 with identical link weights, that the values of the nodal control centrality are distributed symmetrically. For certain node (e.g., 1 or 155), the chain is fully controllable with a single input signal. For majority of the nodes, the control centrality measure is less than *N*. In fact, we can show analytically that the control centrality value of each node is given by (see Supplementary Note 2)
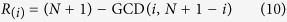
where GCD(*m*, *n*) is the greatest common divisor of the positive integers *m* and *n*. Figure 2(b) shows, the distribution of the control centrality values versus the network size *N*. Two clusters of periodic behavior of *R*_(*i*)_ present as *N* is increased. The periodic phenomena can be verified in terms of Eq. (10).

The combination of the two clusters of periodic behavior lead to the emergence of complex control centrality in undirected chains. Let num(*R*) be the number of the distinct control centrality values in a chain with a certain size. The dependence of num(*R*) on *N* is shown in Fig. 2(c). Analytically, num(*R*) can be determined from the following equation where, for fixed *N*, num(*R*) is the total number of all integer solutions of *f*_*a*_ and *f*_*b*_ that satisfy *f*_*a*_ · *f*_*b*_ = *N* + 1 (see Supplementary Note 2):
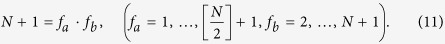


Thus, the solution of num(*R*) is related with prime numbers, accounting for the complex result of num(*R*) in a simple chain structure. Specifically, if *N* + 1 is a prime number, there is only one integer solution of Eq. (11): *f*_*a*_ = 1 and *f*_*b*_ = *N* + 1, leading to num(*R*) = 1 [the hollow circles in Fig. 2(c)]. When *N* + 1 is the square of a prime number, the integer solutions are (*f*_*a*_, *f*_*b*_) = (1, *N* + 1) and (*f*_*a*_, *f*_*b*_) = 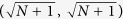, accounting for num(*R*) = 2, as shown by the red circles in Fig. 2(c). For num(*R*) > 2, the situation will become more complicated, because of the inequality of exchanging *f*_*a*_ and *f*_*b*_. For example, if *N* + 1 is the product of two different prime number, num(*R*) will be 3. A typical case is *N* + 1 = 6, for which there are three integer solutions: (*f*_*a*_, *f*_*b*_) = (1, 6), (*f*_*a*_, *f*_*b*_) = (2, 3) and (*f*_*a*_, *f*_*b*_) = (3, 2). However, the scenario that *N* + 1 is cube of a prime number can result in num(*R*) = 3 as well. For instance, when *N* + 1 = 2^3^ = 8, there are three integer solutions: (*f*_*a*_, *f*_*b*_) = (1, 8), (*f*_*a*_, *f*_*b*_) = (2, 4) and (*f*_*a*_, *f*_*b*_) = (4, 2). As a result, Fig. 2(c) exhibits rich behavior of num(*R*) as the length *N* of an undirected chain is increased.

For an undirected chain graph with random weights, the control centrality is simpler than that of a directed chain graph. We can as well offer theoretical results (see Supplementary Note 2). Specifically, when *N* = 2*n* + 1 is odd, we have
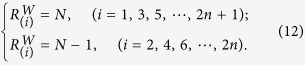
When *N* = 2*n* is even, we have
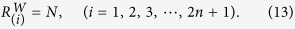


The results are graphically shown in Fig. 2(d), which differs from the results in Fig. 2(c) with identical weights. The fact that random weights eliminates lots of linear correlations in the network matrix accounts for the simplification of the control centrality.

For undirected regular graphs with identical weights, the eigenvalues can be calculated analytically^10^,^47^,^48^, so can be *R*_(*i*)_, as listed in Table 1. We see that the control centrality of the star network is either 2 or 3, and that of the fully connected network is 2, regardless of the network size. For a ring network, almost half of the network can be controlled by a single input. The results for chain networks are relatively more complicated, for which the control centrality values are symmetrically distributed, which can be obtained from Eq. (10) (see Supplementary Note 2). In general, the control centrality 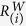 of the undirected regular graphs with random weights is simpler than that of the undirected regular graphs with identical weights, because of the elimination of linear correlation by random weights (see Table 1).

**Figure 3:**
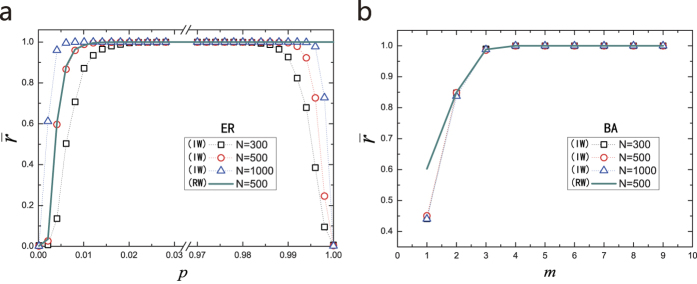
Control centrality of undirected model networks. (**a**) Average control centrality 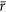 versus the connecting probability *p* for Erdös-Rényi (ER) random networks. (**b**) 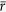 versus half of the average degree *m* = 〈*k*〉/2 for Barabási-Albert (BA) scale-free networks. IW and RW represent identical link weights and random link weights, respectively. All the networks are undirected with symmetric coupling matrices. The data points are averaged over 50 independent network realizations. The representative network sizes are *N* = 300, 500 and 1000.

Figure 3(a) shows the control centrality versus a key structural parameter, the connecting probability, for undirected Erdös-Rényi (ER) random networks with identical link weights. We see that, regardless of the network size, in the regime of small values of the connecting probability *p*, the value of 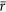 increases monotonically with *p*, indicating that making the network more dense can on average enhance the control efficacy. However, in the extreme regime where *p* is close to unity (e.g., exceeding 0.99), 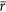 begins to decrease toward zero due to the effect of identical link weights. The control centrality of ER random networks associated with random link weights differs significantly when the network becomes very dense. Specifically, Fig. 3(a) show that 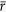 is always one, as *p* approaches unity. The difference is as well attributed to the elimination of linear correlation by random weights, but such effect of random is negligible in sparse ER network.

Figure 3(b) shows, for Barabási-Albert (BA) scale free networks, 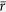 versus half of the average degree *m* = 〈*k*〉/2, where we see that 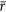 increases rapidly toward unity as 〈*k*〉 is increased, regardless of the number of new links associated with the addition of a new node into the network during its growing process. We see that, qualitatively similar to ER networks, making a scale free network more densely connected can enhance its control efficacy. We also see that because of the general sparsity of the BA network, random link weights have negligible effect on 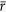 for *m* ≤ 2 compared to identical link weights.

**Figure 4:**
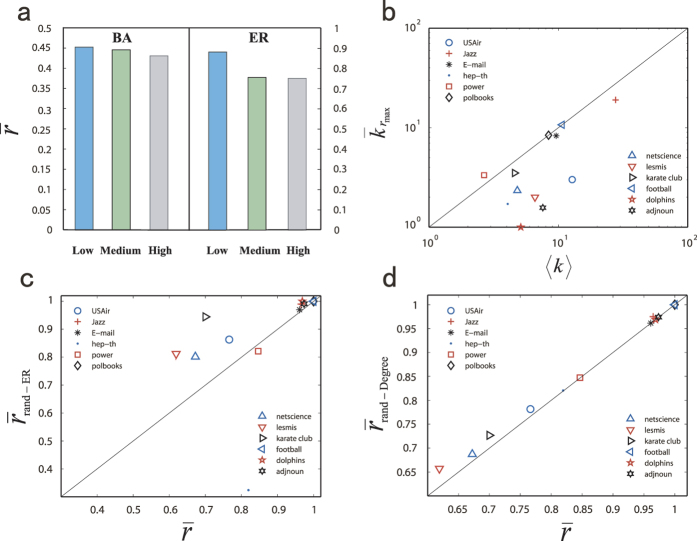
Role of hubs on control centrality in model and empirical networks. (**a**) The average control centrality (bars) for the low-, medium- and high-degree nodes in ER and SA networks of size *N* = 500 and average degree 〈*k*〉 = 2, where the control centrality of hubs is generally less than that for smaller degree nodes. The results are averaged over 500 network realizations. For the ER networks, different connected components are considered separately. (**b**) Mean degree of the nodes with the maximum control centrality *r*_*max*_ as compared with the mean degree of all nodes for a number of empirical networks. It can be seen that for these real-world networks the nodes with relatively large values of the control centrality are not hub nodes, which is consistent with the results in (**a**). (**c**) For randomized empirical networks and (**d**) for the randomized networks but with the degrees preserved, the values of 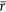 in comparison with these from the original networks.

We characterize the control efficacy for a number of real world (empirical) networks. The results are listed in Table 2. (For the empirical networks with random weights, its corresponding control centrality are slightly higher than the origin network topology.) An issue is whether the hub nodes carry a stronger control centrality in undirected networks. We find that the average control centrality of hub nodes is generally smaller than that of the other nodes in undirected networks, which is consistent with the finding that driver nodes avoid hubs in directed networks^49^. To demonstrate this counterintuitive phenomenon, we divide the nodes into three groups in terms of their degrees: low, medium and high. Figure 4(a) shows, for model ER and BA networks, that the control centrality is generally higher for low-degree nodes than that for the hubs. Figure 4(b) shows the mean degree of the nodes with the maximum control centrality versus the mean degree 〈*k*〉 of all nodes, for each empirical network in Table 2. We see that the values of 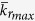, the degree value at which maximum control efficacy is achieved, are significantly smaller than or comparable to 〈*k*〉, indicating the nodes with large values of control centrality are generally not hubs. To provide further evidence for the determining role of nodal degree in the control efficacy, we randomize each empirical network by converting it into an ER random network, keeping the network size *N* and its diameter *L* unchanged. As shown in Fig. 4(c), for some networks there is no correlation between the values of 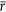 for the original and randomized networks, indicating that the full randomization process has effectively eliminated any topological features of the original network that determine the control efficacy. We then apply a degree-preserving procedure^4^,^50^,^51^ that randomly rewires the links but keeps the degree of each node unchanged. Contrary to the case of full randomization [Fig. 4(c)], when the nodal degrees are preserved, there is little change in the value of 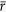, indicating strongly that degree is the key characteristic that determines the control efficacy.

### Identification of controllable nodes

For an arbitrary undirected network, given a control input matrix, we can obtain the dimension of the controllable subspace by calculating the control efficacy. An issue of practical importance is how to identify the actual set of nodes that can be controllable, i.e., the set of controllable nodes for a given control input configuration. Here, we offer a general method based on network diffusion dynamics to address this issue. Specifically, note that the *N* × *Nm* controllability matrix 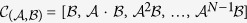 can be expressed iteratively as
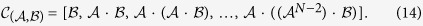


**Figure 5:**
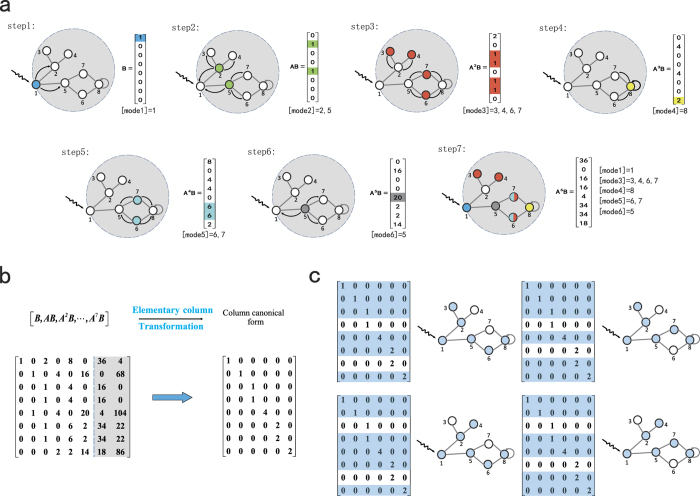
Illustration of our method to identify the set of controllable nodes. Take as an example a simple undirected network with self-loops. (**a**) A step by step illustration of the diffusion process over the network from the driver node, where a control signal is applied at node 1. The newly appeared mode at each step is marked with different colors. At step 7, the iteration column vector 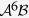 can be expressed as a linear combination of the former modes, so the corresponding value of the control efficacy is 6. (**b**) For the controllability matrix 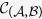, its column canonical form generated by the elementary column transformation. For a fixed value of the control efficacy measure *r*, the column canonical form can be performed only for *r* iterations of the column vector. (**c**) There is a one-to-one correspondence between the controllable nodes and the rows that are linearly dependent upon others in the column canonical form. In the specific case shown, there are four distinct configurations of the controllable nodes (marked in blue). Nevertheless, the number of controllable nodes is fixed and solely determined by 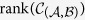.

For the *N* × *N* matrix 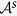, between any pair of nodes (e.g., *i* and *j*), there exists a path of length *s*:
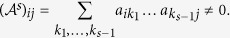


Regarding the nonzero elements of 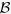 as sources of diffusion, the controllability matrix 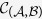 can be viewed as being formed by a diffusion process from the nodes with control matrix 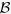 to all the controllable nodes in the network in (*N* − 1) time steps, generating the corresponding diffusion mode for each column of 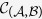. At time step *s*, the matrix product 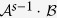 is a linear combination of the mode at the *s* step and the modes from all prior forward steps. The rank of 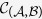 is determined by the number of the distinct modes of diffusion. In general, unless 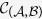 has a full rank, there is interdependence among its columns. Using this fact, we can prove that, for fixed 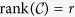, the distinct diffusion modes are fully contained in the former *r* iteration steps (Supplementary Note 3). Consequently, we can implement the following elementary column transformation on the controllability matrix to obtain
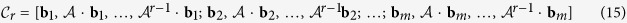
so that the controllable nodes correspond to the maximal linearly independent group of the rows. To illustrate the method explicitly, we present a concrete example, as shown in Fig. 5, where the diffusion process can be seen by noting the newly appeared diffusion mode (color marked) at each time step. We next perform the elementary column transformation on 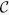 to obtain its column canonical form that reveals the linear dependence among the rows, where the rows that are linearly independent of other rows correspond to the controllable nodes. Note that the configuration of drivers is not unique as it depends on the order with which the elementary transform is implemented. While there are many possible choices of the linearly dependent rows, the number of controllable nodes is fixed and determined solely by 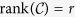. Our procedure of finding the driver nodes is rigorous, as guaranteed by our theory of control efficacy and the column canonical form associated with the matrix rank.

## Discussion

For complex networks in the real world, from the standpoint of control not every node is externally accessible. Often, control signals can be applied to a limited set of nodes or just a few nodes. If the network structure is known, theoretically it is possible to determine a specific set of nodes to apply the control signals, e.g., through identification of maximum matching in SCT. However, the set of control nodes so determined may not overlap with the set of externally accessible nodes. Under these circumstances it is not possible to control the whole network. Nonetheless, there are situations where full control of the entire network is not necessary. A fundamental question is then, if control is applied to a few nodes or even a single node, what fraction of the network can be controlled? That is, for a complex network of arbitrary structure, what is the control efficacy or, equivalently, the dimension of the controllable subspace of the underlying network?

The issue of control efficacy (or control centrality if control is applied at a single node) was addressed in a previous work^36^ but for directed networks. The contribution of the present paper is a rigorous framework based on the theory of exact controllability^10^,^14^ to determine, for undirected complex networks of arbitrary structure (regular, random, or scale-free, weighted or unweighted, with or without self loops, etc.), their control efficacy. From the mathematical control theory, the control efficacy is given by the rank of the Kalman controllability matrix, the determination of which is computationally prohibitive for large networks. Utilizing the non-singular similarity transformation, we discovered a mathematical theorem that enables us to convert rank calculation into a counting problem in terms of the block matrices associated with the distinct eigenvalues of the network coupling matrix. The framework allows us to determine, rigorously, the control efficacy of not only model complex networks, but also a large number of real world networks. Physically, we developed the picture of diffusion, i.e., to view the control process as a signal originated from the driver node and diffused through the controllable subnetwork. The powerful combination of rigorous mathematical theorem and physical reasoning leads to the discovery of striking phenomena in controlling complex networks. For example, more densely connected networks in general have stronger control efficacy, regardless of their topology, and nodal degree is key to control efficacy. However, hub nodes in general have low values of control centrality as compared with majority of the nodes in the network.

From the perspective of fundamental science, our framework of control efficacy represents an important step forward in understanding, quantitatively, the controllability of complex networks at the detailed level of individual nodes. (Extension of our control efficacy framework to analyzing the efficacy of observability of complex networks is straightforward - see Supplementary Note 5). Practically, our theory provides a method and algorithms that can be used to identify efficiently the nodes that possess the strongest possible control centrality. This can have significant applications. For example, given a social network, our framework allows the nodes with the largest control efficacy, i.e., the nodes that can control the largest possible fraction of the network, to be identified. Similarly, for a complex infrastructure network, we can determine a small set of critical nodes to obtain maximum possible control of the network to achieve the highest possible energy efficiency.

Despite our initial success as reported in the present paper, many outstanding issues remain. For example, in real world networks the estimated link weights are not exactly known, which will lead to errors in determining the control efficacy. A mathematical uncertainty or error analysis is needed, but at the present a rigorous treatment seems difficult. Also, our framework of control efficacy relies on complete knowledge of the network structure. What if there is missing information about nodes, links and/or their weights? - at the present we do not have a theory to deal with this practically important issue. Last but not least, our entire theory is based on hypothesizing the underlying complex network as a linear and time invariant dynamical systems. Although much effort has been dedicated to controlling complex networks with nonlinear dynamics^52^,^53^,^54^, a general approach for measuring control efficacy remains to be an outstanding problem^55^. The main challenge stems from the fact that the control efficacy is determined by both network structure and dynamics, in contrast to the network governed by linear dynamics. Much further effort is called for in the extremely rapidly developing field of controlling complex networks.

## Methods

For an undirected network with arbitrary link weights [Eq. (1)], the matrix 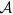 is symmetric and so is diagonalizable: there exists an orthogonal matrix 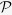 and a diagonal matrix 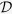 such that 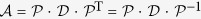 with 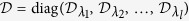, where *λ*_*i*_’s (*i* = 1, …, *l*) are the distinct eigenvalues of 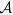 and 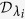 is the diagonal block matrix of 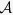 associated with *λ*_*i*_. The size of 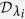 is given by the multiplicity of *λ*_*i*_. We write
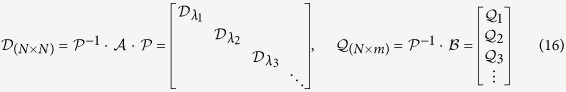


For a linear dynamical system, its controllability is invariant under any non-singular transform. The control efficacy of the original system can then be determined by calculating the rank of the transformed Kalman matrix 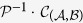 [Eq. (3)].

### Single control input

When the system is subject to a single control input, the control matrix 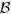 and the transformed control matrix 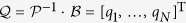 is an *N* × 1 column vector. If 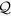 has zero element, the corresponding row in the the transformed Kalman matrix 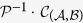 is zero. For the nonzero elements in 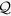, the corresponding eigenvalues can be of two types.

(i) Case I: Distinct eigenvalues. An illustrative example for this case is shown in Fig. 1(a), where the values of *q*_1_, *q*_2_ and *q*_3_ are assumed to be nonzero, corresponding to the eigenvalues *λ*_1_, *λ*_2_ and *λ*_3_, respectively. The corresponding row of (*q*_1_, *q*_2_, *q*_3_) in 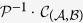 is a Vandermonde matrix, whose rows are linearly independent. In this case, the rank of the controllability matrix is nothing but the number of the nonzero elements corresponding to distinct eigenvalues of 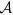.

(ii) Case II: Degenerate eigenvalues. When there is eigenvalue multiplicity, the rows of 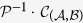 are linearly dependent upon each other. An example of the controllability matrix is
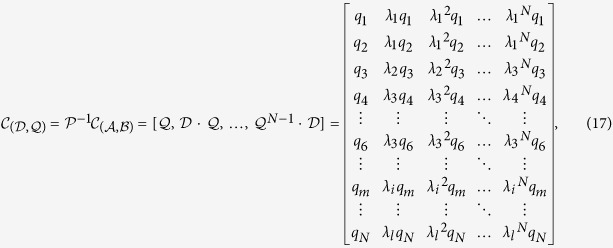
where the rows of *q*_4_ and *q*_6_ are linearly dependent. If the linearly dependent rows in 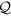 have nonzero elements, they together contribute one to the rank of the controllability matrix.

For a single control input, the calculation of the rank of 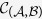 is thus equivalent to counting the corresponding nonzero elements in 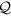. We have
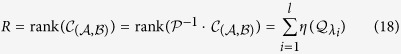


Since the control matrix 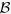 has a single column, the control centrality of the input node is given by
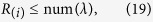
where the num(*λ*) is the number of the distinct eigenvalues of 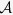.

### Multiple control inputs

With multiple control input signals, the transformed control matrix 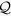 has the dimensional *N* × *m*. To illustrate our method of rank calculation explicitly, we consider the first two columns in 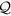. The matrices 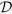 and 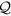 can be written as
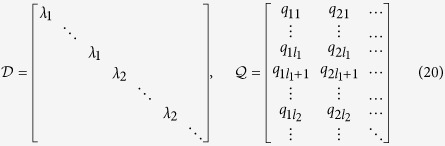


Adjusting the order of the original column vectors appropriately, we can convert the transformed Kalman matrix 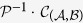 into a form in which two single controller inputs are applied sequentially, i.e.,
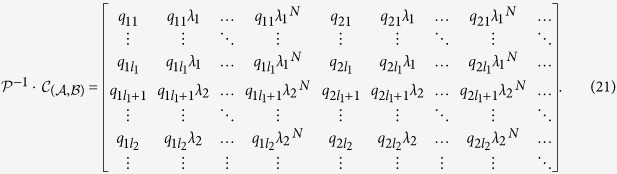
For the case where the control matrix 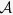 has distinct eigenvalues, if certain rows of the transformed control matrix 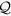 contain nonzero elements, the corresponding rows of the transformed Kalman matrix 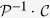 must be linearly independent of each other. The matrix 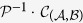 can be organized into a block matrix form, where each block corresponds to one distinct eigenvalue and its dimension is the multiplicity of the eigenvalue. The rank of such a matrix is the sum of the rank values of the sub-block matrices. In particular, letting the algebraic multiplicity of the eigenvalue *λ*_1_ be *l*_1_, we have
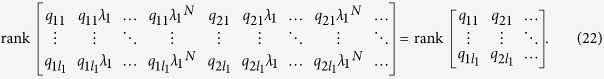
In general, for multiple control inputs, the control efficacy 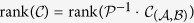 is the sum of the rank values of the sub-block matrices:
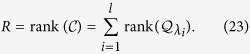


## Additional Information

**How to cite this article**: Gao, X.-D. *et al*. Control efficacy of complex networks. *Sci. Rep.* **6**, 28037; doi: 10.1038/srep28037 (2016).

## Supplementary Material

Supplementary Information

## Figures and Tables

**Table 1:** The distinct eigenvalues, their numbers num(*λ*), and the nodal control centrality for regular undirected graphs.

**Network**	**Eigenvalues**	**num(*****λ***)	***R***_(***i***)_	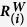
Star network	0(*N* − 2), 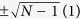	3	*R*_(1)_ = 2, *R*_(*i*)_ = 3, (*i* = 2, …, *N*)	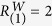 , 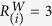 , (*i* = 2, …, *N*)
Fully connected network	*N* − 1(1), −1(*N* − 1)	2	*R*_(*i*)_ = 2, (*i* = 1, …, *N*)	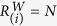 , (*i* = 1, …, *N*)
Ring network	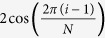	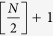	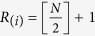 , (*i* = 1, …, *N*)	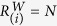 , (*i* = 1, …, *N*)
Undirected chain	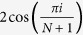	*N*	*R*_(*i*)_ = (*N* + 1) − GCD(*i*, *N* + 1 − *i*), (*i* = 1, …, *N*)	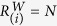 or *N* − 1

Here, 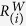 is associated with random link weights and the other variables are associated with identical weights. The algebraic multiplicity of the eigenvalues is indicated for the star and fully connected networks.

**Table 2:** Control centrality of empirical networks.

Data Sets	Nodes	Edges	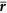	*r*_max_	*r*_min_	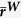	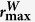	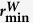
Adjnoun^56^	112	425	0.97377	0.98214	0.97321	0.97481	0.98214	0.97321
Dolphins^57^	62	159	0.96878	0.98387	0.96774	0.97633	0.98387	0.96774
Football^58^	115	615	1	1	1	1	1	1
karate club^59^	34	78	0.69983	0.73529	0.67647	0.80536	0.82353	0.79412
Lesmis^60^	77	254	0.61899	0.63636	0.61039	0.84702	0.85714	0.84416
Netscience^56^	1589	2742	0.04060	0.16174	0.00063	0.07580	0.26306	0.00063
Polbooks^61^	105	441	1	1	1	1	1	1
Power^24^	4941	6594	0.84098	0.84739	0.80935	0.87344	0.88241	0.82372
Hep-th^62^	8361	15751	0.39915	0.57218	0.00012	0.45460	0.65184	0.00012
Email^63^	1133	5451	0.96125	0.96204	0.96028	0.96163	0.96293	0.96117
Jazz^64^	198	2742	0.96500	0.96970	0.96464	0.96740	0.96970	0.96465
USAir^65^	332	2126	0.76639	0.77410	0.76205	0.76944	0.77410	0.76506

For each network, its size, the total number of links, the average value, its maximum and minimum values for identical link weights and random link weights, are given. The structural data of all the networks are available online (see Supplementary Table 1). Superscript *W* represents random weights.
