# Supplementary material for: Control efficacy of complex networks
Source: Sci Rep. 2016 Jun 21;6:28037. doi: 10.1038/srep28037 (PMC4914948; doi:10.1038/srep28037)
Supplement: Supplementary Information [file srep28037-s1.pdf]

Supplementary Information for

Control efficacy of complex networks

Xin-Dong Gao, Wen-Xu Wang, and Ying-Cheng Lai

Contents

1 Supplementary Table 1 **2**

2 Supplementary Note 1: Illustration of rank calculation **3**

3 Supplementary Note 2: Control centrality of simple regular graphs **5**

4 Supplementary Note 3: Identification of controllable nodes **11**

5 Supplementary Note 4: Method of exploiting controllable subspace **12**

6 Supplementary Note 5: Efficacy of observability of complex networks **16**

1 Supplementary Table 1

Table 1. Summary of the real networks analyzed in Table 2 in the main text. The details of the real-world undirected networks studied in this paper. Type column indicates unweighted (UW) or weighted (W) networks. The quantities N and E denote the network size and the number of links, respectively. The last column is the description of the networks. The structural data of all the networks are available online.

Data Sets	Type	N	E	Description
Adjnoun [1]	UW	112	425	Adjacency network of common adjectives and nouns in Dickens' novel David Copperfield
Dolphins [2]	UW	62	159	An undirected social network of frequent associations among 62 dolphins
Football [3]	UW	115	615	American football games among Division IA colleges during regular season Fall 2000
Karate club [4]	UW	34	78	Social network of friendship of a karate club at a US university in the 1970s
Lesmis [5]	UW	77	254	Coappearance network of characters in the novel Les Miserables
Netscience [1]	UW	1589	2742	Coauthorship network of scientists working on network theory and experiment
Polbooks [6]	UW	105	441	A network of books on US politics published around 2004 presidential election and sold on Amazon
Power [7]	UW	4941	6594	A network characterizing the topology of the Western United States Power Grid
Hep-th [8]	W	8361	15751	Network of coauthorship among scientists posting preprints on the High-Energy Theory e-Print Archive
Email [9]	UW	1133	5451	Interchanges among members of the University Rovira i Virgili (Tarragona)
Jazz [10]	UW	198	2742	Links of the network of Jazz musicians
USAir [11]	W	332	2126	US Air flight network, 1997

2 Supplementary Note 1: Illustration of rank calculation

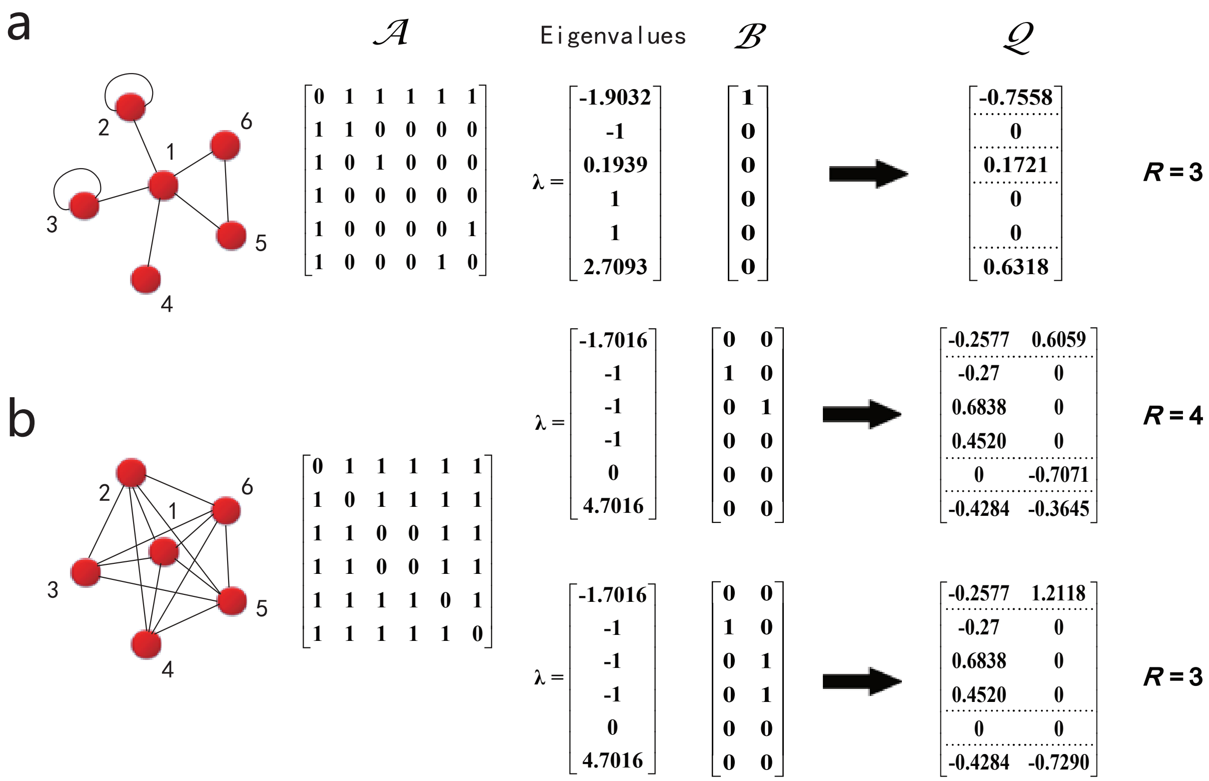

Figure 1. Illustration of our method to calculate the rank and the control efficacy. (a) A simple undirected network with self-loops and (b) a relatively densely connected undirected network. For each case, the adjacency matrix $\mathcal{A}$, its eigenvalues, the control input matrix $\mathcal{B}$, and the transformed control matrix $\mathcal{Q}$ are given. The rows in $\mathcal{Q}$ are divided into distinct sub-blocks as indicated by the dotted lines, where each block corresponds to a distinct eigenvalue of $\mathcal{A}$. For the network in (a), the number of nonzero elements in each block is 3, so $R = 3$. For each network in (b), the rank of the controllability matrix $\mathcal{C}_{(\mathcal{A},\mathcal{B})}$ is equal to the sum of the ranks of all the sub-blocks. The rank values for the two networks in (b) are 4 and 3, respectively.

To illustrate our method to calculate the rank of the controllability matrix $\mathcal{C}_{(\mathcal{A},\mathcal{B})}$ in an explicit and transparent manner, we present some simple examples, as shown in Fig. 1. For each network, we compute the eigenvalues λ_i of the network coupling (adjacency) matrix $\mathcal{A}$. For each control input matrix $\mathcal{B}$, we obtain the transformed control matrix $\mathcal{Q}$ corresponding to each distinct eigenvalue. Our theory of control efficacy gives that the rank $\mathcal{C}_{(\mathcal{A},\mathcal{B})}$ is determined by the nonzero elements in $\mathcal{Q}$. For the networks in Fig. 1(a), according to Eq. (4) in the main text, we have

$$\text{rank}(\mathcal{C}_{(\mathcal{A},\mathcal{B})}) = \eta(\mathcal{Q}_{-1.90132}) + \eta(\mathcal{Q}_{-1}) + \eta(\mathcal{Q}_{0.1939}) + \eta(\mathcal{Q}_{1}) + \eta(\mathcal{Q}_{2.7093}) = 1 + 0 + 1 + 0 + 1 = 3.$$

For the networks in Fig. 1(b), there are two types of control input. For the control input matrix $\mathcal{B}$ with two unity elements, we have

$$\mathrm{rank}(\mathcal{C}_{(\mathcal{A},\mathcal{B})}) = \mathrm{rank}(\mathcal{Q}_{-1.7016}) + \mathrm{rank}(\mathcal{Q}_{-1}) + \mathrm{rank}(\mathcal{Q}_{0}) + \mathrm{rank}(\mathcal{Q}_{4.7016}) = 1 + 1 + 1 + 1 = 4.$$

For the other case, we have

$$\mathrm{rank}(\mathcal{C}_{(\mathcal{A},\mathcal{B})}) = \mathrm{rank}(\mathcal{Q}_{-1.7016}) + \mathrm{rank}(\mathcal{Q}_{-1}) + \mathrm{rank}(\mathcal{Q}_{0}) + \mathrm{rank}(\mathcal{Q}_{4.7016}) = 1 + 1 + 0 + 1 = 3.$$

We thus see that, for the networks in Fig. 1(b), more nonzero elements in the control input matrix $\mathcal{B}$ do not necessarily lead to a higher rank values or control efficacy.

3 Supplementary Note 2: Control centrality of simple regular graphs

According to the Lemma in Supplementary Note 4, we analytically determine the control efficacy for four regular, unweighted and undirected networks: a star graph, a fully connected network, a ring, and a chain.

Star graph. For a star graph of N nodes, its coupling matrix is:

$$\mathcal{A} = \begin{bmatrix} 0 & 1 & 1 & \dots & 1 \\ 1 & 0 & 0 & \dots & 0 \\ 1 & 0 & 0 & \dots & 0 \\ \vdots & \vdots & \vdots & \ddots & \vdots \\ 1 & 0 & 0 & \dots & 0 \end{bmatrix}, \tag{2-1}$$

The eigenvalues and the associated algebraic multiplicity $\delta(\lambda)$ are: $\lambda_1 = \sqrt{N-1}, \delta(\lambda_1) = 1$; $\lambda_2 = -\sqrt{N-1}, \delta(\lambda_2) = 1$; $\lambda_3 = 0, \delta(\lambda_3) = N-2$ [12, 13]. We can prove the following theorem:

Theorem (Control centrality of a star graph): Given a star graph of N nodes and a single control signal, the control efficacy of the central node is 2 and that of any leaf node is 3.

Proof: We label the central node as node 1 and the leaf nodes as $2, \dots, N$. For an arbitrary nonzero eigenvalue $\lambda = \pm\sqrt{N-1}$ and a single control signal applied at node i, the augmented matrix can be simplified through elementary transformation as

$$\begin{matrix} (\mathcal{A}^{\mathrm{T}} - \lambda\mathcal{I}) \cdot \mathbf{x}_- = 0 \\ \mathcal{B}^{\mathrm{T}} \cdot \mathbf{x}_- = 0 \end{matrix} \Leftrightarrow \begin{bmatrix} -\lambda & 1 & \dots & 1 & \dots & 1 \\ 1 & -\lambda & & & & \\ \vdots & & \ddots & & & \\ 1 & & & -\lambda & & \\ \vdots & & & & \ddots & \\ 1 & & & & & -\lambda \\ 0 & 0 & \dots & 1 & \dots & 0 \end{bmatrix} \rightarrow \begin{bmatrix} 0 & 0 & \dots & 0 & \dots & 0 \\ 0 & -\lambda & & & & \\ \vdots & & \ddots & & & \\ 1 & & & 0 & & \\ \vdots & & & & \ddots & \\ 0 & & & & & -\lambda \\ 0 & 0 & \dots & 1 & \dots & 0 \end{bmatrix} \tag{2-2}$$

The rank of the above matrix is N. We thus have

$$\mathrm{rank}\mathcal{Q}_\lambda = \mathrm{rank}[\lambda\mathcal{I} - \mathcal{A}, \mathcal{B}] - \mathrm{rank}[\lambda\mathcal{I} - \mathcal{A}] = N - (N-1) = 1.$$

We see that the contribution of each single eigenvalue λ to the control efficacy is one.

For the zero eigenvalue with multiplicity $N-2$, the control efficacy depends on the specific node at which the control signal is applied. For the center-node, the augmented matrix is

$$\begin{matrix} (\mathcal{A}^{\mathrm{T}} - 0\mathcal{I}) \cdot \mathbf{x}_- = 0 \\ \mathcal{B}^{\mathrm{T}} \cdot \mathbf{x}_- = 0 \end{matrix} \Leftrightarrow \begin{bmatrix} 0 & 1 & \dots & 1 & \dots & 1 \\ 1 & 0 & & & & \\ \vdots & & \ddots & & & \\ 1 & & & 0 & & \\ \vdots & & & & \ddots & \\ 1 & & & & & 0 \\ 1 & 0 & \dots & 0 & \dots & 0 \end{bmatrix} \rightarrow \begin{bmatrix} 0 & 1 & \dots & 0 & \dots & 0 \\ 1 & 0 & & & & \\ \vdots & & \ddots & & & \\ 0 & & & 0 & & \\ \vdots & & & & \ddots & \\ 0 & & & & & 0 \\ 1 & 0 & \dots & 0 & \dots & 0 \end{bmatrix}. \tag{2-3}$$

We then have $\text{rank}(\mathcal{Q}_0) = \text{rank}[0 \cdot \mathcal{I} - \mathcal{A}, \mathcal{B}] - \text{rank}[0\mathcal{I} - \mathcal{A}] = 2 - 2 = 0$, i.e., the null eigenvalue contributes nothing to the control efficacy. Analogously, when controlling any leaf node, we have $\text{rank}[0\mathcal{I} - \mathcal{A}, \mathcal{B}] - \text{rank}[0\mathcal{I} - \mathcal{A}] = \text{rank}\mathcal{Q}_0 = 3 - 2 = 1$. That is, a control input at each leaf node increases the control efficacy by one. We conclude that, in general, for a star graph, the control efficacy for the central node is 2 and that of any leaf node is 3. ■

Fully connected network. The coupling matrix of a fully connected network of N nodes is:

$$\mathcal{A} = \begin{bmatrix} 0 & 1 & 1 & \dots & 1 \\ 1 & 0 & 1 & \dots & 1 \\ 1 & 1 & 0 & \dots & 1 \\ \vdots & \vdots & \vdots & \ddots & \vdots \\ 1 & 1 & 1 & \dots & 0 \end{bmatrix} \tag{2-4}$$

Its eigenvalues and the respective algebraic multiplicities are: $\lambda_1 = N - 1, \delta(\lambda_1) = 1$ and $\lambda_2 = -1, \delta(\lambda_2) = N - 1$ [12, 13].

Theorem: For a fully connected network of N nodes, the control centrality of any node is 2.

Proof: For a single control signal at node i and for the eigenvalue $N - 1$ (with multiplicity one), the augmented matrix is

$$\begin{array}{l} (\mathcal{A}^{\mathrm{T}} - (N-1)\mathcal{I}) \cdot \mathbf{x}_- = 0 \\ \mathcal{B}^{\mathrm{T}} \cdot \mathbf{x}_- = 0 \end{array} \Leftrightarrow \begin{bmatrix} 1-N & 1 & \dots & 1 & \dots & 1 \\ 1 & 1-N & \dots & 1 & \dots & 1 \\ \vdots & \vdots & \ddots & \vdots & \ddots & \vdots \\ 1 & 1 & \dots & 1-N & \dots & 1 \\ \vdots & \vdots & \ddots & \vdots & \ddots & \vdots \\ 1 & 1 & \dots & 1 & \dots & 1-N \\ 0 & 0 & \dots & 1 & \dots & 0 \end{bmatrix}$$

$$\to \begin{bmatrix} 1 & -1 & & & & \\ & 1 & -1 & & & \\ & & \ddots & \ddots & & \\ & & & 1 & -1 & \\ & & & & 1 & -1 \\ 0 & 0 & \dots & 0 & \dots & 0 \\ 0 & 0 & \dots & 1 & \dots & 0 \end{bmatrix}. \tag{2-5}$$

The above augmented matrix has rank N, so we have $\text{rank}\mathcal{Q}_{N-1} = \text{rank}[(N-1)\mathcal{I} - \mathcal{A}, \mathcal{B}] - \text{rank}[(N-1)\mathcal{I} - \mathcal{A}] = N - (N-1) = 1$. The contribution of node i to the control efficacy is one.

For the eigenvalue -1 with multiplicity $N-1$, for a single control input at node i, we have

$$\begin{array}{l}(\mathcal{A}^{\mathrm{T}}+\mathcal{I})\cdot\mathbf{x}_{-}=0\\ \mathcal{B}^{\mathrm{T}}\cdot\mathbf{x}_{-}=0\end{array} \Leftrightarrow \begin{bmatrix} 1 & 1 & \dots & 1 & \dots & 1 \\ 1 & 1 & \dots & 1 & \dots & 1 \\ \vdots & \vdots & \ddots & \vdots & \ddots & \vdots \\ 1 & 1 & \dots & 1 & \dots & 1 \\ \vdots & \vdots & \ddots & \vdots & \ddots & \vdots \\ 1 & 1 & \dots & 1 & \dots & 1 \\ 0 & 0 & \dots & 1 & \dots & 0 \end{bmatrix} \rightarrow \begin{bmatrix} 1 & 1 & \dots & 1 & \dots & 1 \\ 0 & 0 & \dots & 0 & \dots & 0 \\ \vdots & \vdots & \ddots & \vdots & \ddots & \vdots \\ 0 & 0 & \dots & 0 & \dots & 0 \\ \vdots & \vdots & \ddots & \vdots & \ddots & \vdots \\ 0 & 0 & \dots & 0 & \dots & 0 \\ 0 & 0 & \dots & 1 & \dots & 0 \end{bmatrix} \tag{2-6}$$

We have $\text{rank}\mathcal{Q}_1 = \text{rank}[\mathcal{I}-\mathcal{A},\mathcal{B}] - \text{rank}[\mathcal{I}-\mathcal{A}] = 2-1 = 1$, so node i with a single control signal at it contributes one to the control efficacy. ■

Cycle graph. For a cycle graph of N nodes, the coupling matrix is

$$\mathcal{A} = \begin{bmatrix} 0 & 1 & 0 & \dots & 1 \\ 1 & 0 & 1 & \dots & 0 \\ 0 & 1 & 0 & \dots & 0 \\ \vdots & \vdots & \vdots & \ddots & \vdots \\ 1 & 0 & 0 & \dots & 0 \end{bmatrix}. \tag{2-7}$$

The eigen-spectrum of a cycle graph is [12,13]: $\lambda_i = 2\cos(2\pi(i-1)/N)$ for $i=1,\dots,N$.

Theorem For a cycle graph of N nodes, the controlling centrality of every node is $R_{(i)} = [N/2]+1$ for $i=1,\dots,N$.

Proof: First, we prove that, when controlling a single node, the rank of $[\lambda I - A, B]$ is increased by one as compared with the case of no control.

Say we apply a control signal at node i. For an arbitrary eigenvalue λ, the augmented matrix is:

$$\begin{array}{l}(\mathcal{A}^{\mathrm{T}}-\lambda\mathcal{I})\cdot\mathbf{x}_{-}=0\\ \mathcal{B}^{\mathrm{T}}\cdot\mathbf{x}_{-}=0\end{array} \Leftrightarrow \begin{bmatrix} -\lambda & 1 & 0 & \dots & 1 \\ 1 & -\lambda & 1 & \dots & 1 \\ 0 & 1 & -\lambda & \dots & 0 \\ \vdots & \vdots & \vdots & \ddots & \vdots \\ 1 & 0 & 0 & \dots & -\lambda \\ 0 & 0 & 1 & \dots & 0 \end{bmatrix} \tag{2-8}$$

Supposing when a row vector $\mathcal{B}^{\mathrm{T}}$ is added to the augmented matrix, its rank is unchanged. Thus, the row vector $\mathcal{B}^{\mathrm{T}}$ can be linearly represented by the row vectors of $(\mathcal{A}^{\mathrm{T}}-\lambda\mathcal{I})$. For instance, when we control node 2, solutions exist for the linear system $(\mathcal{A}^{\mathrm{T}}-\lambda\mathcal{I})\cdot\mathbf{x} = \mathbf{b}_{(2)}$. We have

$$(\mathcal{A}^{\mathrm{T}}-\lambda\mathcal{I})\cdot\mathbf{x}_{-} = \mathbf{b}_{(2)} \Leftrightarrow \begin{bmatrix} -\lambda & 1 & 0 & \dots & 1 \\ 1 & -\lambda & 0 & \dots & 1 \\ 0 & 1 & -\lambda & \dots & 1 \\ \vdots & \vdots & \vdots & \ddots & \vdots \\ 1 & 0 & 0 & \dots & -\lambda \end{bmatrix} \begin{bmatrix} x_1 \\ x_2 \\ x_3 \\ \vdots \\ x_N \end{bmatrix} = \begin{bmatrix} 0 \\ 1 \\ 0 \\ \vdots \\ 0 \end{bmatrix}. \tag{2-9}$$

We define the following permutation transform:

$$\begin{bmatrix} 1 & 2 & 3 & 4 & \dots & N \\ N & 1 & 2 & 3 & \dots & N-1 \end{bmatrix} \Rightarrow \begin{array}{l} \bar{x}_1 = x_N \\ \bar{x}_2 = x_1 \\ \bar{x}_3 = x_2 \\ \bar{x}_4 = x_3 \\ \vdots \\ \bar{x}_N = x_{N-1}. \end{array} \tag{2-10}$$

There are solutions for the linear system: $(\mathcal{A}^{\mathrm{T}} - \lambda\mathcal{I}) \cdot \bar{\mathbf{x}} = \mathbf{b}_{(3)}$:

$$\begin{bmatrix} -\lambda & 1 & 0 & \dots & 1 \\ 1 & -\lambda & 0 & \dots & 1 \\ 0 & 1 & -\lambda & \dots & 1 \\ \vdots & \vdots & \vdots & \ddots & \vdots \\ 1 & 0 & 0 & \dots & -\lambda \end{bmatrix} \begin{bmatrix} \bar{x}_1 \\ \bar{x}_2 \\ \bar{x}_3 \\ \vdots \\ \bar{x}_N \end{bmatrix} = \begin{bmatrix} 0 \\ 0 \\ 1 \\ \vdots \\ 0 \end{bmatrix}. \tag{2-11}$$

Through a series of similar permutation transformations, we can represent an arbitrary row vector as a linear combination of the row vectors of the matrix $(\mathcal{A}^{\mathrm{T}} - \lambda\mathcal{I})$, which can be transformed into a unitary matrix by implementing elementary row transformations. This means that the rank of $(\mathcal{A}^{\mathrm{T}} - \lambda\mathcal{I})$ is N. There seems to be a contradiction between the rank N and the characteristic property of the matrix $(\mathcal{A}^{\mathrm{T}} - \lambda\mathcal{I})$ for the eigenvalue λ. Consequently, for a cycle graph, when controlling a single node, the row vector $\mathcal{B}^{\mathrm{T}}$ and any row vectors of the matrix $(\mathcal{A}^{\mathrm{T}} - \lambda\mathcal{I})$ are linearly dependent. For any eigenvalue λ, the rank of the matrix $[\lambda\mathcal{I} - \mathcal{A}, \mathcal{B}]$ is increased by one as compared with the case of no control input.

Next, we note that the eigenvalue distribution of the cycle graph depends on its size. For odd size $N = 2n + 1$, there is a single eigenvalue 2 and n distinct eigenvalues with multiplicity 2. For even size $N = 2n$, there are two single eigenvalues: 2, -2 and $n - 1$ distinct eigenvalues with multiplicity 2. For single node control, the control efficacy of the cycle graph is then given by:

$$\begin{aligned} &N = 2n + 1(\text{odd}) : R_{(i)} = n + 1, (i = 1, \dots, N); \\ &N = 2n(\text{even}) : R_{(i)} = n + 1, (i = 1, \dots, N). \end{aligned} \tag{2-12}$$

■

Undirected chain graph. The coupling matrix of an undirected chain network of N nodes is

$$\mathcal{A} = \begin{bmatrix} 0 & 1 & & & \\ 1 & 0 & 1 & & \\ & \ddots & \ddots & \ddots & \\ & & 1 & 0 & 1 \\ & & & 1 & 0 \end{bmatrix}, \tag{2-13}$$

its eigenvalues are $\lambda_i = 2\cos[\pi i/(N+1)]$ for $i = 1, \ldots, N$, and the algebraic multiplicity of each distinct eigenvalue [12, 13] is $\delta(\lambda_i) = 1$ for $i = 1, \ldots, N$. A chain network can be controlled simply by driving a single node at either end, so we have $R_{(1)} = R_{(N)} = N$, which holds for weighted undirected chain graphs, too [14, 15].

Theorem: Given an undirected chain graph of N nodes, the control centrality $R_{(i)}$ of each node is determined by

$$R_{(i)} = (N+1) - \mathrm{GCD}(i, N+1-i) \tag{2-14}$$

where $\mathrm{GCD}(m, n)$ is the greatest common divisor of the two positive integers (m and n).

Proof: We define a matrix $P_N \in \mathbb{R}^{N\times N}$ and a column vector $\mathbf{b}_{(i)} \in \mathbb{R}^{1\times N}$ as

$$\mathcal{P}_{N(N\times N)} = \begin{bmatrix} 0 & 1 & & & \\ 1 & 0 & 1 & & \\ & \ddots & \ddots & \ddots & \\ & & 1 & 0 & 1 \\ & & & 1 & 0 \end{bmatrix} \text{ and } \mathbf{b}_{(i)} = \begin{bmatrix} 0 \\ \vdots \\ 1 \\ \vdots \\ 0 \end{bmatrix}. \tag{2-15}$$

When we control node i, the rank of the controllability matrix $\mathcal{C}$ is $r < N$. Using our Lemma in Supplementary Note 4, we see that there exist $N - r$ vectors v that satisfy $\mathcal{A}^{\mathrm{T}} \cdot \mathbf{v} = \lambda \mathbf{v}$ and $\mathbf{b}_{(i)}{}^{\mathrm{T}} \cdot \mathbf{v} = 0$. Equivalently, this means that there exist corresponding eigenvectors v of A with $\mathbf{v} = [v_1, 0, v_2]$ for $v_1 \in \mathbb{R}^{i-1}$ and $v_2 \in \mathbb{R}^{N-i}$. In terms of the components, we have

$$\mathcal{P}_{i-1} \cdot \mathbf{v}_1 = \lambda \mathbf{v}_1, \quad (\mathbf{v}_1)_{i-1} + (\mathbf{v}_2)_1 = 0, \quad \text{and} \mathcal{P}_{N-i} \cdot \mathbf{v}_2 = \lambda \mathbf{v}_2, \tag{2-16}$$

where $(\mathbf{v}_1)_{i-1}$ is the $(i-1)$th element of the column vector $\mathbf{v}_1$ and $(\mathbf{v}_2)_1$ is the first element of the column vector $\mathbf{v}_2$. To satisfy the first condition in Eq. (2-16), there are two cases: (i) $\mathbf{v}_1 = 0$ and (ii) $\mathbf{v}_1$ and λ are the eigenvector and eigenvalue of $\mathcal{P}_{i-1}$, respectively. If $\mathbf{v}_1 = \mathbf{0}$, then $(\mathbf{v}_1)_{i-1} = 0$ and $(\mathbf{v}_2)_1 = 0$. We can verify that the first and last components of any eigenvector of $\mathcal{P}_{i-1}$ are nonzero. Thus we have $\mathbf{v}_2 = 0$ and $\mathbf{v} = 0$, contradicting the definition of the eigenvector $\mathbf{v}$ that it must be nonzero.

For case (ii), if $\mathcal{P}_{i-1}$ and $\mathcal{P}_{N-i}$ have at least one common eigenvalue, say λ_0, with corresponding eigenvectors $\mathbf{v}_{10}$ and $\mathbf{v}_{20}$, respectively, we get $\mathbf{v} = [v_{10}, 0, kv_{20}]$ and $\lambda = \lambda_0$, which satisfy Eq. (2-16), where k is a scaling factor given by $(v_{10})_{i-1} + k(v_{20})_1 = 0$.

We thus see that, if $\mathcal{P}_{i-1}$ and $\mathcal{P}_{N-i}$ have $N - r$ common eigenvalues, the rank of $\mathcal{C}$ is r. Note that the eigenvalues of $\mathcal{P}_{i-1}$ and $\mathcal{P}_{N-i}$ are given by $\lambda_m = 2\cos(\pi m/i)$ (for $m = 1, \ldots, i-1$)) and $\lambda_n = 2\cos[\pi n/(N-i+1)]$ (for $n = 1, \ldots, N-i$), respectively. The number of common eigenvalues is given by $\mathrm{GCD}(i, N+1-i)$. As a result, the control centrality $R_{(i)}$ of any node in the chain is

$$R_{(i)} = (N+1) - \mathrm{GCD}(i, N+1-i). \tag{2-17}$$

Let $\mathrm{num}(R)$ be the number of distinct control centrality in the network, which is determined by the integer factors of $N+1$, denoted as f_a, and let f_b be the number of the integer factors. Taking advantage of the symmetry of the path control centrality: $f_a \in (1, \ldots, [N/2]+1)$ and $f_a \in (2, \ldots, N)$, we can calculate $\mathrm{num}(R)$ through the number of all the integer solutions of the

following equation:

$$N+1=f_a\cdot f_b,\ (f_a=1,\dots,[\frac{N}{2}]+1, f_b=2,\dots,N) \tag{2-18}$$

The Control centrality of an undirected chain graph with random weights is given by when $N=2n+1$(odd),

$$\begin{cases} R^W_{(i)}=N,\ (i=1,3,5,\cdots,2n+1); \\ R^W_{(i)}=N-1,\ (i=2,4,6,\cdots,2n); \end{cases} \tag{2-19}$$

when $N=2n$(even),

$$R^W_{(i)}=N,\ (i=1,2,3,\cdots,2n+1). \tag{2-20}$$

Proof: If the chain size $N=2n+1$ is odd, when we only directly control node i, the control signal diffuse from the driven node to the whole network by step. According to the topological structure of chain network, the diffusion modes consist of odd node and even node alternately. When imposing input signal at even node, without loss of generality we set i=4 and the single non-zero element to be unity for convenience, then the Kalman controllability matrix C is

$$C=\begin{bmatrix} 0&0&0&*&0&*&0&*&0&\cdots\\ 0&0&*&0&*&0&*&0&*&\cdots\\ 0&*&0&*&0&*&0&*&0&\cdots\\ 1&0&*&0&*&0&*&0&*&\cdots\\ 0&*&0&*&0&*&0&*&0&\cdots\\ 0&0&*&0&*&0&*&0&*&\cdots\\ 0&0&0&*&0&*&0&*&0&\cdots\\ 0&0&0&0&*&0&*&0&*&\cdots\\ 0&0&0&0&0&*&0&*&0&\cdots\\ \vdots&\vdots&\vdots&\vdots&\vdots&\vdots&\vdots&\vdots&\vdots&\ddots \end{bmatrix} \tag{2-21}$$

where $*$ are random free parameters. We can see the nonzero random elements of the even columns are in the odd rows, thus these even columns are linear independence for all but an exceptional set of values of the free parameters which forms a proper variety with Lebesgue measure zero in the parameter space. The $n+1$ odd columns of C only have nonzero random elements which corresponding to the n even nodes$(2,4,6,\cdots,2n)$, thus the rank of the sub-matrix of odd columns is n. Therefore, for the chain network with $N=2n+1$ nodes, when control even node$(i=2,4,6,\cdots,2n)$, $R^W_{(i)}=N-1$. When we control odd nodes$(i=1,3,5,\cdots,2n+1)$, the nonzero random elements of the even columns are in the even rows, and the nonzero random elements in the odd columns corresponding to the odd nodes. Thus all the columns are linear independence except for a zero measure set.

If the chain size $N=2n$ is even, follow the similar sketch of the proof above, when imposing a control signal at node i, we can get $R^W_{(i)}=N,\ (i=1,2,3,\cdots,2n)$. ■

4 Supplementary Note 3: Identification of controllable nodes

The controllability matrix $\mathcal{C}_{(\mathcal{A},\mathcal{B})}$ can be viewed as the matrix representation of a diffusion process from the control input node (described by the matrix $\mathcal{B}$) in $N-1$ time steps. If $\mathrm{rank}(\mathcal{C}_{(\mathcal{A},\mathcal{B})}) = r$, we can prove that the diffusion process is determined by the matrix $\mathcal{C}_r$ given by

$$\mathcal{C}_r = [\mathcal{B}, \mathcal{A}\cdot\mathcal{B}, \mathcal{A}^2\cdot\mathcal{B}, \ldots, \mathcal{A}^{r-1}\cdot\mathcal{B}]. \tag{3-1}$$

Proof. When the system has a single control input, the matrix $\mathcal{B}$ is reduced to an $N\times 1$ column vector. We write $\mathcal{B} = \mathbf{B}$. If the rank of $\mathcal{C}$ is r, the linear independence columns in $\mathcal{C}$ constitute the first r columns. Otherwise, if the ith column ($i \le r$) in $\mathcal{C}$ can be linearly represented by the prior $(i-1)$ columns, we have

$$\mathbf{C}^i = \mathcal{A}^{i-1}\cdot\mathbf{B} = \alpha_1\mathbf{B} + \alpha_2\mathcal{A}\cdot\mathbf{B} + \alpha_2\mathcal{A}^2\cdot\mathbf{B} + \ldots + \alpha_{i-1}\mathcal{A}^{i-2}\cdot\mathbf{B}, \tag{3-2}$$

where $\mathbf{C}^i$ ($i = 1,\ldots,r$) is the ith column of $\mathcal{C}$. Multiplying the matrix $\mathcal{A}$ from the left, we have

$$\mathbf{C}^{i+1} = \mathcal{A}^{i}\cdot\mathbf{B} = \alpha_1\mathcal{A}\cdot\mathbf{B} + \alpha_2\mathcal{A}^2\cdot\mathbf{B} + \ldots + \alpha_{i-1}\mathcal{A}^{i-1}\cdot\mathbf{B}, \tag{3-3}$$

i.e., the $i+1$th column can be expressed as a linear combination of the $1,\ldots,(i-1)$th columns [Eq. (3-2) and Eq. (3-3)], and all the subsequent columns can be expressed in a similar manner. As a result, the rank of $\mathcal{C}$ is less than or equal to i, contradicting the hypothesis $\mathrm{rank}(\mathcal{C}_{(\mathcal{A},\mathcal{B})}) = r$.

When there are multiple input control signals, i.e., $\mathcal{B} = [\mathbf{b}_1, \mathbf{b}_2, \ldots, \mathbf{b}_m]$ is an $N\times m$ matrix, we have

$$\mathcal{C}_r = [\mathbf{b}_1, \mathbf{b}_2, \ldots, \mathbf{b}_m; \mathcal{A}\cdot\mathbf{b}_1, \mathcal{A}\cdot\mathbf{b}_2, \ldots, \mathcal{A}\cdot\mathbf{b}_m; \mathcal{A}^{r-1}\cdot\mathbf{b}_1, \mathcal{A}^{r-1}\cdot\mathbf{b}_2, \ldots, \mathcal{A}^{r-1}\cdot\mathbf{b}_m] \tag{3-4}$$

Through proper adjustment of the order of the column vectors, we can rewrite the matrix $\mathcal{C}_r$ in terms of the single control inputs as

$$\mathcal{C}_r = [\mathbf{b}_1, \mathcal{A}\cdot\mathbf{b}_1, \ldots, \mathcal{A}^{r-1}\cdot\mathbf{b}_1; \mathbf{b}_2, \mathcal{A}\cdot\mathbf{b}_2, \ldots, \mathcal{A}^{r-1}\cdot\mathbf{b}_2; \ldots; \mathbf{b}_m, \mathcal{A}\cdot\mathbf{b}_m, \ldots, \mathcal{A}^{r-1}\cdot\mathbf{b}_m]. \tag{3-5}$$

If $\mathcal{C}_r$ does not contain all the r linearly independent columns, all the columns in the submatrices $\mathcal{A}^{r-1}\cdot\mathcal{B}$ $[\mathcal{A}^{r-1}\cdot\mathcal{B}, \ldots, \mathcal{A}^{N-1}\cdot\mathcal{B}]$ are linearly dependent, contradicting the hypothesis $\mathrm{rank}(\mathcal{C}_{(\mathcal{A},\mathcal{B})}) = r$. ■

5 Supplementary Note 4: Method of exploiting controllable subspace

If the system is not fully controllable, i.e., if the state space is not filled with controllable states $\mathbf{x}_+$, it can be decomposed as a direct sum: $\mathbf{X} = \mathbf{X}_c{}^+ \oplus \mathbf{X}_c{}^-$, where $\mathbf{X}_c{}^+$ stands for the controllable subspace spanned by the column vectors of the controllability matrix $\mathcal{C}$, and $\mathbf{X}_c{}^-$ is the complementary subspace [16–18]. In principle, using the orthogonality property, we can identify the nodes in the uncontrollable subspace by solving $\mathbf{x}_-^{\mathrm{T}} \cdot \mathcal{C} = 0$. However, it is computationally difficult to solve this equation directly. We prove the following lemma, which can be exploited to overcome the difficulty.

Lemma: For an arbitrarily network with diagonalizable matrix $\mathcal{A}$, the equations $\mathbf{x}_-^{\mathrm{T}} \cdot [\mathcal{A} - \lambda\mathcal{I}, \mathcal{B}] = 0$ and $\mathbf{x}_-^{\mathrm{T}} \cdot \mathcal{C} = 0$ are equivalent, for any eigenvalue λ of $\mathcal{A}$.

proof: If $\mathbf{x}_-^{\mathrm{T}} \cdot [\mathcal{A} - \lambda\mathcal{I}, \mathcal{B}] = 0$, then $\mathbf{x}_-^{\mathrm{T}} \cdot \mathcal{A} = \lambda \mathbf{x}_-^{\mathrm{T}}, \mathbf{x}_-^{\mathrm{T}} \cdot \mathcal{B} = 0$. We have

$$\begin{aligned} \mathbf{x}_-^{\mathrm{T}} \cdot \mathcal{B} &= 0, \\ \mathbf{x}_-^{\mathrm{T}} \cdot \mathcal{A} \cdot \mathcal{B} &= \lambda \mathbf{x}_-^{\mathrm{T}} \cdot \mathcal{B} = 0, \\ &\ldots, \\ \mathbf{x}_-^{\mathrm{T}} \cdot \mathbf{A}^{n-1} \cdot \mathcal{B} &= \lambda^{n-1} \mathbf{x}_-^{\mathrm{T}} \cdot \mathcal{B} = 0, \end{aligned}$$

which means $\mathbf{x}_-^{\mathrm{T}} \cdot [\mathcal{B}, \mathcal{A} \cdot \mathcal{B}, \mathcal{A}^2 \cdot \mathcal{B}, \ldots, \mathcal{A}^{n-1} \cdot \mathcal{B}] = \mathbf{x}_-^{\mathrm{T}} \cdot \mathcal{C} = 0$. Thus $\mathbf{x}_-^{\mathrm{T}}$ is an uncontrollable state and $\mathbf{x}_-^{\mathrm{T}} \cdot \mathcal{C} = 0$ if $\mathbf{x}_-^{\mathrm{T}} \cdot [\mathcal{A} - \lambda\mathcal{I}, \mathcal{B}] = 0$.

Applying the non-singular transformations $\mathbf{y} = \mathcal{P}^{-1} \cdot \mathbf{x}$ and $\mathcal{Q} = \mathcal{P}^{-1} \cdot \mathcal{B}$, we can express the system in a diagonalizable form, which has the same degree of controllability as the original system in the sense that

$$\mathrm{rank}[\lambda\mathcal{I} - \mathcal{A}, \mathcal{B}] = \mathrm{rank}[\lambda\mathcal{I} - \mathcal{D}, \mathcal{Q}], \tag{4-1}$$

with $\mathrm{rank}(\mathcal{B}) = \mathrm{rank}(\mathcal{Q})$. The rank of $[\lambda\mathcal{D} - \mathcal{Q}]$ is determined by the rank of the sub-block matrices composed of the corresponding rows in the transformed control matrix $\mathcal{Q} = \mathcal{P}^{-1} \cdot \mathcal{B}$ for every eigenvalue of $\mathcal{A}$. We have

$$\mathrm{rank}[\lambda\mathcal{I} - \mathcal{A}, \mathcal{B}] - \mathrm{rank}[\lambda\mathcal{I} - \mathcal{A}] = \mathrm{rank}\mathcal{Q}_\lambda \tag{4-2}$$

for any eigenvalue λ, where $\mathcal{Q}_\lambda$ is the submatrix in $\mathcal{Q}$ associated with λ. We have

$$\mathrm{rank}(\mathcal{C}) = \sum_\lambda \mathrm{rank}(\mathcal{Q}_\lambda) = \sum_\lambda (\mathrm{rank}[\lambda\mathcal{I} - \mathcal{A}, \mathcal{B}] - \mathrm{rank}[\lambda\mathcal{I} - \mathcal{A}]) = \sum_\lambda \{\mathrm{rank}[\lambda\mathcal{I} - \mathcal{A}, \mathcal{B}] - [N - \delta(\lambda)]\}, \tag{4-3}$$

where $\delta(\lambda)$ is the algebraic multiplicity of the eigenvalue λ. We then have

$$\sum_\lambda (N - \mathrm{rank}[\lambda\mathcal{I} - \mathcal{A}, \mathcal{B}]) = \sum_\lambda (\delta(\lambda) - \mathrm{rank}(\mathcal{Q}_\lambda) = N - \mathrm{rank}(\mathcal{C}), \tag{4-4}$$

which means that the solution spaces $\mathbf{x}_-^{\mathrm{T}} \cdot [\mathcal{A} - \lambda\mathcal{I}, \mathcal{B}] = 0$ (for any eigenvalue) and $\mathbf{x}_-^{\mathrm{T}} \cdot \mathcal{C} = 0$ have the same dimension. Consequently, for an arbitrarily undirected network, the conditions $\mathbf{x}_-^{\mathrm{T}} \cdot [\mathcal{A} - \lambda\mathcal{I}, \mathcal{B}] = 0$ (for any eigenvalue) and $\mathbf{x}_-^{\mathrm{T}} \cdot \mathcal{C} = 0$ are equivalent. ■

The method of exploiting controllable subspace can be used to identify the uncontrollable states. Since the eigen-subspaces of an undirected network or a diagonalizable matrix are mutually orthogonal, we can reduce the computational effort further by verifying the condition $\mathbf{x}_-^{\mathrm{T}} \cdot [\mathcal{A} - \lambda\mathcal{I}, \mathcal{B}] = 0$ for the required eigenvalue [19], which is effectively equivalent to the following equations:

$$\begin{aligned} (\mathcal{A}^{\mathrm{T}} - \lambda\mathcal{I}) \cdot \mathbf{x}_- &= 0, \\ \mathcal{B}^{\mathrm{T}} \cdot \mathbf{x}_- &= 0. \end{aligned} \tag{4-5}$$

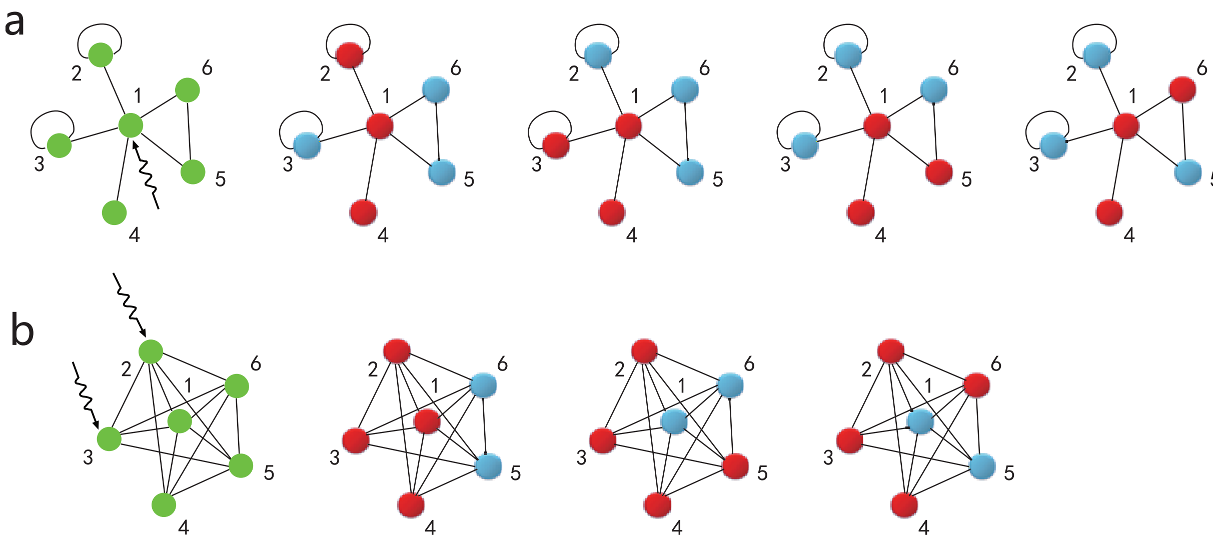

Figure 2. Illustration of the method of exploiting controllable subspace. The wavy arrows indicate the control input and the controllable nodes are marked red. For the network in (a), the four available configurations are (1, 4, 2), (1, 4, 3), (1, 4, 5), (1, 4, 6). The network in (b) permits the following three configurations: (1, 2, 3, 4), (2, 3, 4, 5), (2, 3, 4, 6).

In the following we provide two explicit examples to illustrate the method of exploiting controllable subspace.

For the undirected network in Fig. 2(a), when only node 1 is controlled, the coupling matrix $\mathcal{A}$, its eigenvalues, the original and transformed control matrices are

$$\mathcal{A} = \begin{bmatrix} 0 & 1 & 1 & 1 & 1 & 1 \\ 1 & 1 & 0 & 0 & 0 & 0 \\ 1 & 0 & 1 & 0 & 0 & 0 \\ 1 & 0 & 0 & 0 & 0 & 0 \\ 1 & 0 & 0 & 0 & 0 & 1 \\ 1 & 0 & 0 & 0 & 1 & 0 \end{bmatrix}, \lambda = \begin{bmatrix} -1.9032 \\ -1 \\ -0.1939 \\ 1 \\ 1 \\ 2.7093 \end{bmatrix}, \mathcal{B} = \begin{bmatrix} 1 \\ 0 \\ 0 \\ 0 \\ 0 \\ 0 \end{bmatrix}, \mathcal{Q} = \begin{bmatrix} -0.7558 \\ 0 \\ 0.1721 \\ 0 \\ 0 \\ 0.6318 \end{bmatrix} \tag{4-6}$$

The subspace method stipulates that the modes associated with the eigenvalues 1 and -1 do not depend entirely on the input and, hence, the control efficacy is 3. We solve the augmented equation [Eq. (4-5)] for each eigenvalue. The solutions of the new equations consisting of

the fundamental solutions of the augmented equations represent the controllable states. The procedure is as follows.

$$\begin{array}{l}(\mathcal{A}^{\mathrm{T}} - \mathbf{1}\cdot\mathcal{I})\cdot\mathbf{x}_{-} = 0\\ \mathcal{B}^{\mathrm{T}}\cdot\mathbf{x}_{-} = 0\end{array} \Leftrightarrow \begin{bmatrix} -1 & 1 & 1 & 1 & 1 & 1\\ 1 & 0 & 0 & 0 & 0 & 0\\ 1 & 0 & 0 & 0 & 0 & 0\\ 1 & 0 & 0 & -1 & 0 & 0\\ 1 & 0 & 0 & 0 & -1 & 1\\ 1 & 0 & 0 & 0 & 1 & -1\\ 1 & 0 & 0 & 0 & 0 & 0 \end{bmatrix}\cdot\mathbf{x}_{-} = 0 \tag{4-7}$$

$$\Rightarrow \mathbf{x}_{-} = \begin{bmatrix} 0 & 0\\ -1 & -2\\ 1 & 0\\ 0 & 0\\ 0 & 1\\ 0 & 1 \end{bmatrix} \Rightarrow \begin{array}{l} -x_2 + x_3 = 0\\ -2x_2 + x_5 + x_6 = 0 \end{array}$$

$$\begin{array}{l}(\mathcal{A}^{\mathrm{T}} + \mathbf{1}\cdot\mathcal{I})\cdot\mathbf{x}_{-} = 0\\ \mathcal{B}^{\mathrm{T}}\cdot\mathbf{x}_{-} = 0\end{array} \Leftrightarrow \begin{bmatrix} 1 & 1 & 1 & 1 & 1 & 1\\ 1 & 2 & 0 & 0 & 0 & 0\\ 1 & 0 & 2 & 0 & 0 & 0\\ 1 & 0 & 0 & 1 & 0 & 0\\ 1 & 0 & 0 & 0 & 1 & 1\\ 1 & 0 & 0 & 0 & 1 & 1\\ 1 & 0 & 0 & 0 & 0 & 0 \end{bmatrix}\cdot\mathbf{x}_{-} = 0 \Rightarrow \mathbf{x}_{-} = \begin{bmatrix} 0\\ 0\\ 0\\ 0\\ -1\\ 1 \end{bmatrix} \Rightarrow -x_5 + x_6 = 0 \tag{4-8}$$

The new equations as the constraint condition of the controllable states are

$$\begin{cases} -x_2 + x_3 = 0\\ -2x_2 + x_5 + x_6 = 0\\ -x_5 + x_6 = 0 \end{cases} \tag{4-9}$$

The under-determined system of equations is composed of the states of nodes 2, 3, 5 and 6, whose fundamental solution set is one dimensional. Apparently, the states of nodes 1 and 4 are free with respect to the constraint condition, so they are always controllable. The configuration of the controllable nodes is not unique as it depends on the fundamental solution set, but the number of the driver nodes is fixed at 3. Thus, for this network, we have four configurations: (1, 4, 2), (1, 4, 3), (1, 4, 5), and (1, 4, 6), which are marked red in Fig. 2(a).

For the undirected network in Fig. 2(b), the coupling matrix, its eigenvalue, the original and

transformed matrices are, respectively,

$$\mathcal{A}=\begin{bmatrix}0&1&1&1&1&1\\1&0&1&1&1&1\\1&1&0&0&1&1\\1&1&0&0&1&1\\1&1&1&1&0&1\\1&1&1&1&1&0\end{bmatrix},\lambda=\begin{bmatrix}-1.7016\\-1\\-1\\-1\\0\\4.7016\end{bmatrix},\mathcal{B}=\begin{bmatrix}0&0\\1&0\\0&1\\0&0\\0&0\\0&0\end{bmatrix},\mathcal{Q}=\begin{bmatrix}-0.2577&0.6059\\-0.2794&0\\0.6838&0\\0.4520&0\\0&-0.7071\\-0.4284&-0.3645\end{bmatrix}. \tag{4-10}$$

The original control input matrix $\mathcal{B}$ has the dimension 6×2. The control efficacy is 4. Our theory of control subspace stipulates that only the mode associated with eigenvalue -1 is not entirely determined by the input. Solving the fundamental solution set of the augmented equations, we obtain the controllable states, as follows.

$$\begin{matrix}(\mathcal{A}^{\mathrm{T}}+1\times\mathcal{I})\cdot\mathbf{x}_{-}=0\\ \mathcal{B}^{\mathrm{T}}\cdot\mathbf{x}_{-}=0\end{matrix}\Leftrightarrow\begin{bmatrix}1&1&1&1&1&1\\1&1&1&1&1&1\\1&1&1&0&1&1\\1&1&0&1&1&1\\1&1&1&1&1&1\\1&1&1&1&1&1\\0&1&0&0&0&0\\0&0&1&0&0&0\end{bmatrix}\cdot\mathbf{x}_{-}=0\Rightarrow \tag{4-11}$$

$$\mathbf{x}_{-}=\begin{bmatrix}-1&-1\\0&0\\0&0\\0&0\\1&0\\0&1\end{bmatrix}\Rightarrow\begin{matrix}-x_1+x_5=0\\-x_1+x_6=0\end{matrix}$$

Following a similar procedure to analyze the network in Fig. 2(a), we obtain that the states of nodes 2, 3 and 4 are free with respect to the constraint condition, so they are controllable. The configuration of the controllable nodes is not unique: the three possible configurations are (1, 2, 3, 4), (2, 3, 4, 5), and (2, 3, 4, 6).

6 Supplementary Note 5: Efficacy of observability of complex networks

Due to the duality of controllability and observability, similar to the development of the control efficacy framework, we can define the observability efficacy of complex networks to quantify one's ability to observe the whole system, i.e., to infer the state of the whole system. In particular, consider a complex network system in the presence of observers described by

$$\begin{aligned} \dot{\mathbf{x}} &= \mathcal{A} \cdot \mathbf{x} + \mathcal{B} \cdot \mathbf{u}, \\ \mathbf{y} &= \widetilde{\mathcal{C}} \cdot \mathbf{x}, \end{aligned} \tag{5-1}$$

where $\widetilde{\mathcal{C}}$ is the $r \times N$ observability matrix. For initial state $\mathbf{x}(t_0) = \bar{\mathbf{x}}$, if the output response of the system $\mathbf{y}(t)$ is 0 within the finite time interval $[t_0, t_1]$, the state $\bar{\mathbf{x}}$ is unobservable within $[t_0, t_1]$ and we denote it as $\mathbf{x}_-$. The system described Eq. (5-1) is fully observable if there exists no unobservable state in the state space. We use the symbol ${\mathbf{X}_o}^-$ to denote the set of all unobservable states, which constitute the unobservable subspace of the system, whose complementary space is the observable subspace denoted as ${\mathbf{X}_o}^+$. The rank of the observability matrix $\mathcal{Q}_o$ can be defined as the observability efficacy of the underlying network:

$$\text{rank}(\mathcal{Q}_o) = \text{rank} \begin{bmatrix} \widetilde{\mathcal{C}} \\ \widetilde{\mathcal{C}} \cdot \mathcal{A} \\ \widetilde{\mathcal{C}} \cdot \mathcal{A}^2 \\ \vdots \\ \widetilde{\mathcal{C}} \cdot \mathcal{A}^{N-1} \end{bmatrix}. \tag{5-2}$$

Analogous to controllability, for an undirected network with diagonalizable matrix $\mathcal{A}$, if the observability matrix $\widetilde{\mathcal{C}}$ has the dimension $r \times N$, it can be transformed into $\bar{\mathcal{C}}$ through a non-singular transformation. The rank of $\mathcal{Q}_o$ is determined by the corresponding element value in the transformed matrix $\bar{\mathcal{C}}$ for every distinct eigenvalue. We have

$$\text{rank}(\mathcal{Q}_o) = \sum_{i=1}^{l} \text{rank}(\bar{\mathcal{C}_{\lambda_i}}). \tag{5-3}$$

Supplementary References

1. Newman, M. E. Finding community structure in networks using the eigenvectors of matrices. *Phys. Rev. E* **74**, 036104 (2006).

2. Lusseau, D. *et al.* The bottlenose dolphin community of doubtful sound features a large proportion of long-lasting associations. *Behav. Ecol. Sociobio.* **54**, 396–405 (2003).

3. Girvan, M. & Newman, M. E. Community structure in social and biological networks. *Proc. Nat. Acad. Sci. (USA)* **99**, 7821–7826 (2002).

4. Zachary, W. W. An information flow model for conflict and fission in small groups. *J. Anthrop. Res.* 452–473 (1977).

5. Knuth, D. E., Knuth, D. E. & Knuth, D. E. *The Stanford GraphBase: A Platform for Combinatorial Computing*, vol. 37 (Addison-Wesley Reading, 1993).

6. Ripeanu, M., Foster, I. & Iamnitchi, A. Mapping the gnutella network: Properties of large-scale peer-to-peer systems and implications for system design. *arXiv preprint cs/0209028* (2002).

7. Watts, D. J. & Strogatz, S. H. Collective dynamics of small-world networks. *Nature* **393**, 440–442 (1998).

8. Newman, M. E. The structure of scientific collaboration networks. *Proc. Nat. Acad. Sci. (USA)* **98**, 404–409 (2001).

9. Guimera, R., Danon, L., Diaz-Guilera, A., Giralt, F. & Arenas, A. Self-similar community structure in a network of human interactions. *Phys. Rev. E* **68**, 065103 (2003).

10. Gleiser, P. M. & Danon, L. Community structure in jazz. *Adv. Complex Sys.* **6**, 565–573 (2003).

11. http://vlado.fmf.uni-lj.si/pub/networks/pajek/data/gphs.htm .

12. Brouwer, A. E. & Haemers, W. H. *Spectra of Graphs* (Springer Science & Business Media, 2011).

13. Van Mieghem, P. *Graph Spectra for Complex Networks* (Cambridge University Press, 2010).

14. Yuan, Z., Zhao, C., Di, Z., Wang, W.-X. & Lai, Y.-C. Exact controllability of complex networks. *Nature Commun.* **4** (2013).

15. Parlangeli, G. & Notarstefano, G. On the reachability and observability of path and cycle graphs. *IEEE Trans. Auto. Cont.* **57**, 743–748 (2012).

16. Rugh, W. J. *Linear System Theory*, vol. 2 (prentice hall Upper Saddle River, NJ, 1996).

17. Kailath, T. *Linear Systems*, vol. 1 (Prentice-Hall Englewood Cliffs, NJ, 1980).

18. Antsaklis, P. J. & Michel, A. N. *Linear Systems* (Springer Science & Business Media, 2006).

19. Lay, D. C. *Linear Algebra and Its Applications* (Addison-Wesley Publishing Company, New York, 2003).
